# Supplementary material for: Efficacy of MRI data harmonization in the age of machine learning: a multicenter study across 36 datasets
Source: Sci Data. 2024 Jan 23;11:115. doi: 10.1038/s41597-023-02421-7 (PMC10805868; doi:10.1038/s41597-023-02421-7)
Supplement: Supplementary file 1 — Related Manuscript File [file 41597_2023_2421_MOESM1_ESM.pdf]

Supplementary Information

Table of contents

Supplementary Table 1

Table S1. Description of the demographic characteristics of each single-center dataset. Data from single-center datasets have been included in one specific meta-dataset considering the following age ranges: CHILDHOOD (5 – 13 years), ADOLESCENCE (11 – 20 years), and ADULTHOOD (18 – 87 years) (more details in section 2.1). All single-center datasets have also been included in the LIFESPAN meta-dataset.

**Dataset**	**Institution**	**# participants**	**Females (%)**	**Age range min-max**	**Age median (IQR)**	**Meta-dataset**
ABIDEI-CALTECH	California Institute of Technology	19	21.05	17.0 - 56.2	23.6 (15.85)	ADULTHOOD
ABIDEI-CMU	Carnegie Mellon University	13	23.08	20.0 - 40.0	27.0 (9.0)	
ABIDEI-KKI	Kennedy Krieger Institute	33	27.27	8.07 - 12.77	9.97 (1.54)	CHILDHOOD
ABIDEI-LEUVEN	University of Leuven	35	14.29	12.2 - 29.0	16.6 (7.95)	ADOLESCENCE
ABIDEI-MAX_MUN	Ludwig Maximilians University Munich	33	12.12	7.0 - 48.0	26.0 (9.0)	ADULTHOOD
ABIDEI-NYU	New York University Langone Medical Center	105	24.76	6.47 - 31.78	14.38 (8.99)	ADOLESCENCE
ABIDEI-OHSU	Oregon Health & Science University	15	0	8.2 - 11.99	10.08 (1.31)	CHILDHOOD
ABIDEI-OLIN	Olin Center, Institute of Living at Hartford Hospital	16	12.5	10.0 - 23.0	16.5 (6.25)	ADOLESCENCE
ABIDEI-PITT	University of Pittsburgh School of Medicine	27	14.81	9.44 - 33.24	17.13 (8.3)	ADOLESCENCE
ABIDEI-SBL	Social Brain Lab BCN NeuroImaging Center, University Medical Center Groningen and Netherlands Institute for Neuroscience	15	0	20.0 - 42.0	36.0 (11.5)	ADULTHOOD
ABIDEI-SDSU	San Diego State University	22	27.27	8.67 - 16.88	14.42 (2.45)	ADOLESCENCE

ABIDEI-STANFORD	Stanford University	20	20	7.75 - 12.43	9.41 (2.57)	CHILDHOOD
ABIDEI-TRINITY	Trinity College Dublin	25	0	12.04 - 25.66	15.91 (5.25)	ADOLESCENCE
ABIDEI-UCLA	University of California, Los Angeles	47	12.77	9.21 - 17.79	12.68 (2.16)	ADOLESCENCE
ABIDEI-UM	University of Michigan	77	23.38	8.2 - 28.8	14.8 (5.0)	
ABIDEI-USM	University of Utah School of Medicine	43	0	8.77 - 39.39	19.76 (10.4)	
ABIDEII-BNI_1	Barrow Neurological Institute	29	0	18.0 - 64.0	43.0 (27.0)	ADULTHOOD
ABIDEII-EMC_1	Erasmus University Medical Centre	27	18.52	6.33 - 10.12	8.19 (1.41)	CHILDHOOD
ABIDEII-ETH_1	ETH Zürich	24	0	13.83 - 30.67	24.0 (6.79)	ADULTHOOD
ABIDEII-GU_1	Georgetown University	55	49.09	8.06 - 13.8	10.43 (2.96)	CHILDHOOD
ABIDEII-IP_1	Institut Pasteur and Robert Debré Hospital	34	64.71	8.07 - 46.6	22.12 (18.8)	ADULTHOOD
ABIDEII-IU_1	Indiana University	20	25	19.0 - 37.0	22.0 (4.25)	ADULTHOOD
ABIDEII-KKI_32ch	Kennedy Krieger Institute	45	26.67	8.06 - 12.67	10.27 (1.67)	CHILDHOOD
ABIDEII-KKI_8ch	Kennedy Krieger Institute	110	40	8.02 - 12.9	10.3 (1.68)	CHILDHOOD
ABIDEII-NYU_1	New York University Langone Medical Center	30	6.67	5.89 - 23.81	9.11 (3.12)	CHILDHOOD
ABIDEII-OHSU_1	Oregon Health & Science University	56	51.79	8.0 - 14.0	10.0 (2.25)	CHILDHOOD

ABIDEII-SDSU_1	San Diego State University	25	8	8.1 - 17.7	13.0 (5.2)	ADOLESCENCE
ABIDEII-TCD_1	Trinity College Dublin	21	0	10.25 - 20.0	15.25 (5.25)	ADOLESCENCE
ABIDEII-UCD_1	University of California Davis	14	28.57	12.25 - 17.17	14.75 (2.65)	
ABIDEII-UCLA_1	University of California, Los Angeles	16	31.25	7.76 - 14.09	9.01 (1.43)	CHILDHOOD
ABIDEII-USM_1	University of Utah School of Medicine	16	18.75	11.5 - 36.15	23.78 (10.72)	ADULTHOOD
ICBM	International Consortium for Human Brain Mapping	86	52.33	19.0 - 85.0	44.5 (31.5)	ADULTHOOD
IXI-Guys	Guy's Hospital - London	313	55.59	20.07 - 86.2	53.41 (26.08)	ADULTHOOD
IXI-HH	Hammersmith Hospital - London	181	51.38	20.17 - 81.94	48.05 (28.25)	ADULTHOOD
IXI-IOP	Institute of Psychiatry - London	67	65.67	19.98 - 86.32	36.16 (19.85)	ADULTHOOD
NKI2		73	41.1	6.0 - 17.0	12.0 (6.0)	CHILDHOOD

IQR: interquartile range
